# Supplementary material for: Distinct nociception processing in the dysgranular and barrel regions of the mouse somatosensory cortex
Source: Nat Commun. 2022 Jun 29;13:3622. doi: 10.1038/s41467-022-31272-w (PMC9243138; doi:10.1038/s41467-022-31272-w)
Supplement: Supplementary file 1 — Supplementary Information [file 41467_2022_31272_MOESM1_ESM.pdf]

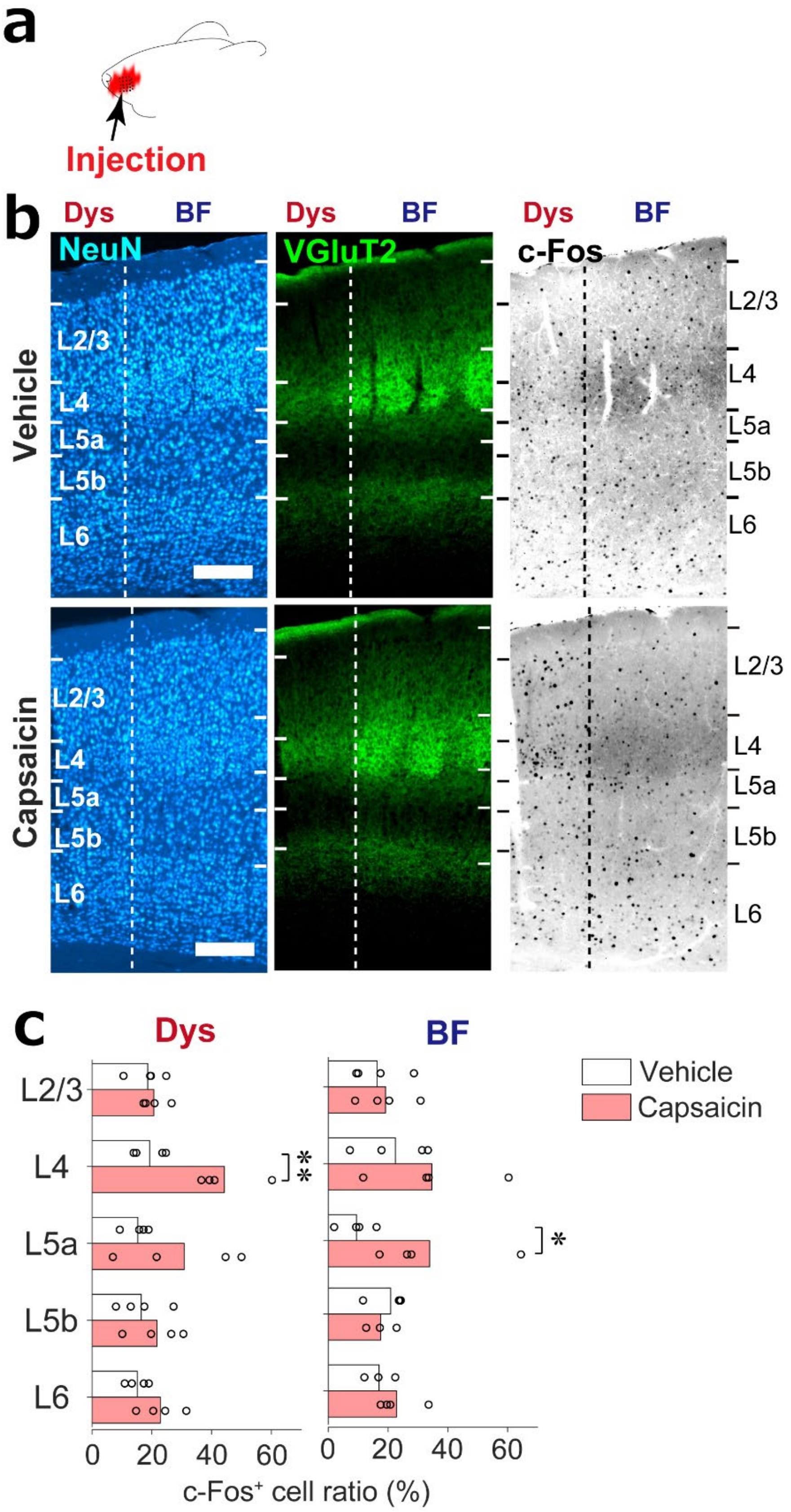
a
Injection
b
Dys
BF
NeuN
VGluT2
c-Fos
Vehicle
Capsaicin
L2/3
L4
L5a
L5b
L6
c
Dys
BF
Vehicle
Capsaicin
0
20
40
60
c-Fos+ cell ratio (%)
*

**Supplementary Fig. 1**

**Dysgranular region in S1 responds to capsaicin injection**

**a**, Vehicle or capsaicin was injected into the left whisker pad. **b**, S1 neurons were activated by injecting capsaicin into the whisker pad. *Left*, NeuN staining to identify neurons. *Middle*, VGluT2 staining to identify the border of Dys and BF (dotted lines). *Right*, c-Fos immunostaining of the same slices. Scale bar, 200 µm. **c**, Capsaicin injection into the whisker pad increased the number of c-Fos-positive neurons in Dys L4 (bar, mean; $**P = 0.0027$, two-way ANOVA followed by Sidak's test) and BF L5a ($*P = 0.0249$). N = 4 animals for capsaicin, and 4 animals for vehicle injection.

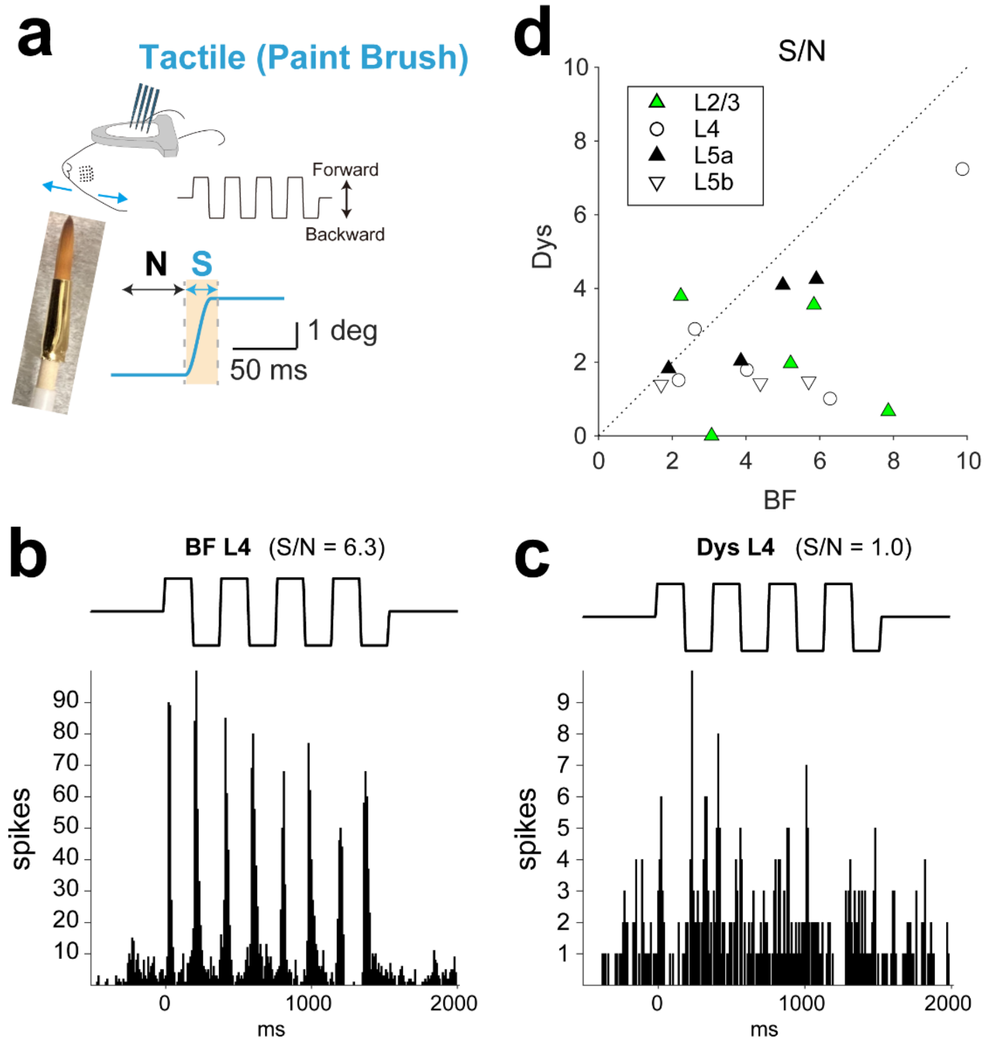


**Supplementary Fig. 2**

**BF responds more selectively than Dys to tactile input into the whisker pad**

**a**, Experimental setup for recording responses to tactile stimulation of the whisker pad. All whiskers and tiny hairs on the left whisker pad were removed so that a paint brush could directly stimulate the whisker pad. The paint brush was moved by a piezo system. **b**, **c**, Examples of PSTHs of MUA to tactile stimuli of BF (b) and Dys (c) neurons. Bin width is 10 ms. **d**, Scatter plot of S/N to tactile stimuli. $P = 0.00074$ (n = 18) by the two-sided Wilcoxon signed-rank test.

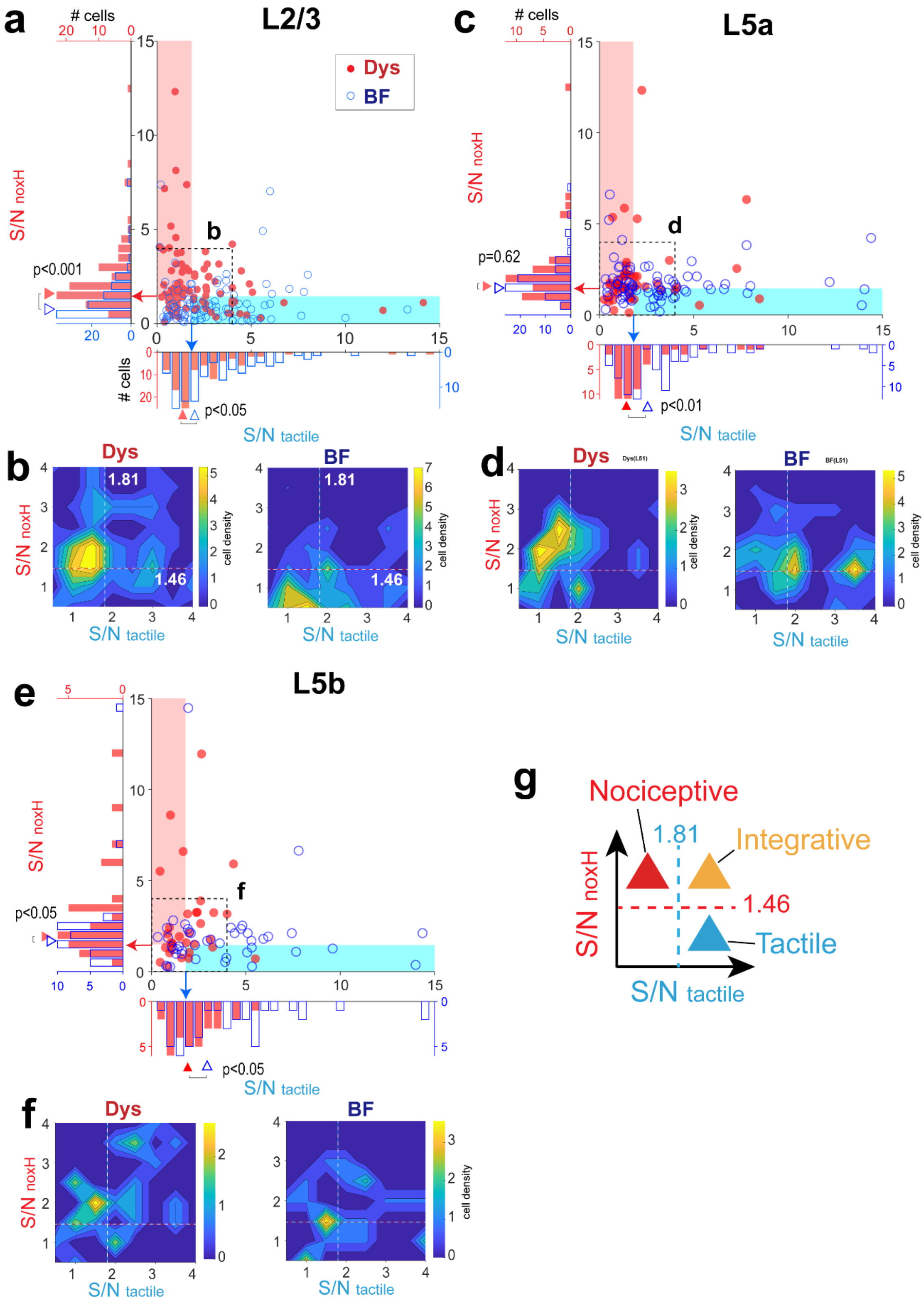


**Supplementary Fig. 3**

**Segregation and integration of noxious heat and tactile information were observed in the**

**S/N scatter plots of all neurons**

**a**, Scatter plot of S/N to a tactile stimulus against S/N to a noxious heat stimulus (noxH) for L2/3 neurons. The neurons in the blue shaded area are the tactile input-preferring neurons, for which the S/N to a tactile stimulus were higher than 1.81 (the median of all neurons, blue arrow) and to noxH were lower than 1.46 (the median of all neurons, red arrow). The neurons in the red shaded area are noxious input-preferring neurons, for which the S/N to a tactile stimulus were lower than 1.81 and to noxH were higher than 1.46. The distribution of S/N for noxH of Dys is significantly higher than that of BF ($P = 4.82 \times 10^{-9}$, two-sided two-sample Kolmogorov–Smirnov test). The red arrowhead on the *y* axis indicates the median S/N for the noxH of Dys neurons, 1.60; the blue arrowhead indicates the median of BF neurons, 0.77. The distribution of S/N for tactile stimuli in Dys is significantly lower than that in BF ($P = 0.012$, two-sided two-sample Kolmogorov–Smirnov test ). The red arrowhead on the *x* axis indicates the median S/N for the tactile stimulation of Dys neurons, 1.36; the blue arrowhead indicates the median of BF neurons, 2.00. **b**, A density map of S/N to tactile stimuli against S/N to noxH for L2/3 neurons, with each S/N <4. **c**, Same as panel *a* but for L5a neurons. The distributions are not significantly different from each other for the S/N of noxH ($P = 0.62$). S/N distributions of tactile stimulations are different from each other ($P = 0.0054$). The arrowheads on the *x* axis indicate the median S/N for the tactile stimulation of Dys (1.43, red) and BF neurons (2.55, blue). The arrowheads on the *y* axis indicate the median S/N for the noxH stimulation of Dys (1.58, red) and BF neurons (1.48, blue). **d**, Same as panel b but for L5a neurons. **e**, The distributions are significantly different from each other for the S/N of noxH ($P = 0.041$, two-sided two-sample Kolmogorov–Smirnov test) and of tactile stimulation ($P = 0.011$, two-sided two-sample Kolmogorov–Smirnov test). The arrowheads on the *x* axis indicate the median S/N for the tactile stimulation of Dys (1.91, red) and BF neurons (2.95, blue). The arrowheads on the *y*-axis indicate the median S/N for the noxH stimulation of Dys (1.92, red) and BF L5b neurons (1.69, blue). **f**, Same as panel *b* but for L5b neurons. **g**, Cell classification (see also Fig. 2c).

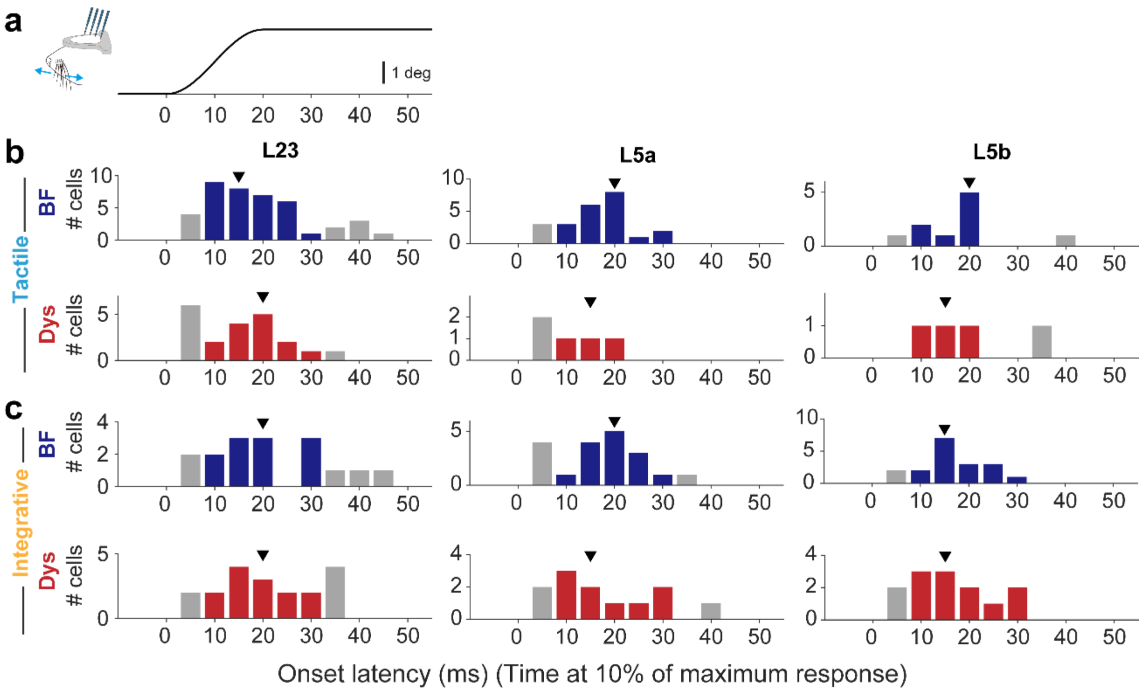


**Supplementary Fig. 4**

**Onset latency to tactile stimuli reflects layer and area specificity.**

**a**, The time course of the whisker stimulus by a piezo device. **b**, The onset latencies of the response to tactile stimulations by tactile cells (see Fig. 3 for the criteria) in each region and layer. The onset latency was calculated as the time at 10% the maximum response to the tactile stimulus. Times smaller than 10 ms or larger than 35 ms (gray bars) are not reasonable as onset latencies[1] and excluded from the calculation of the medians (arrowheads). **c**, Same as b but of integrative cells.

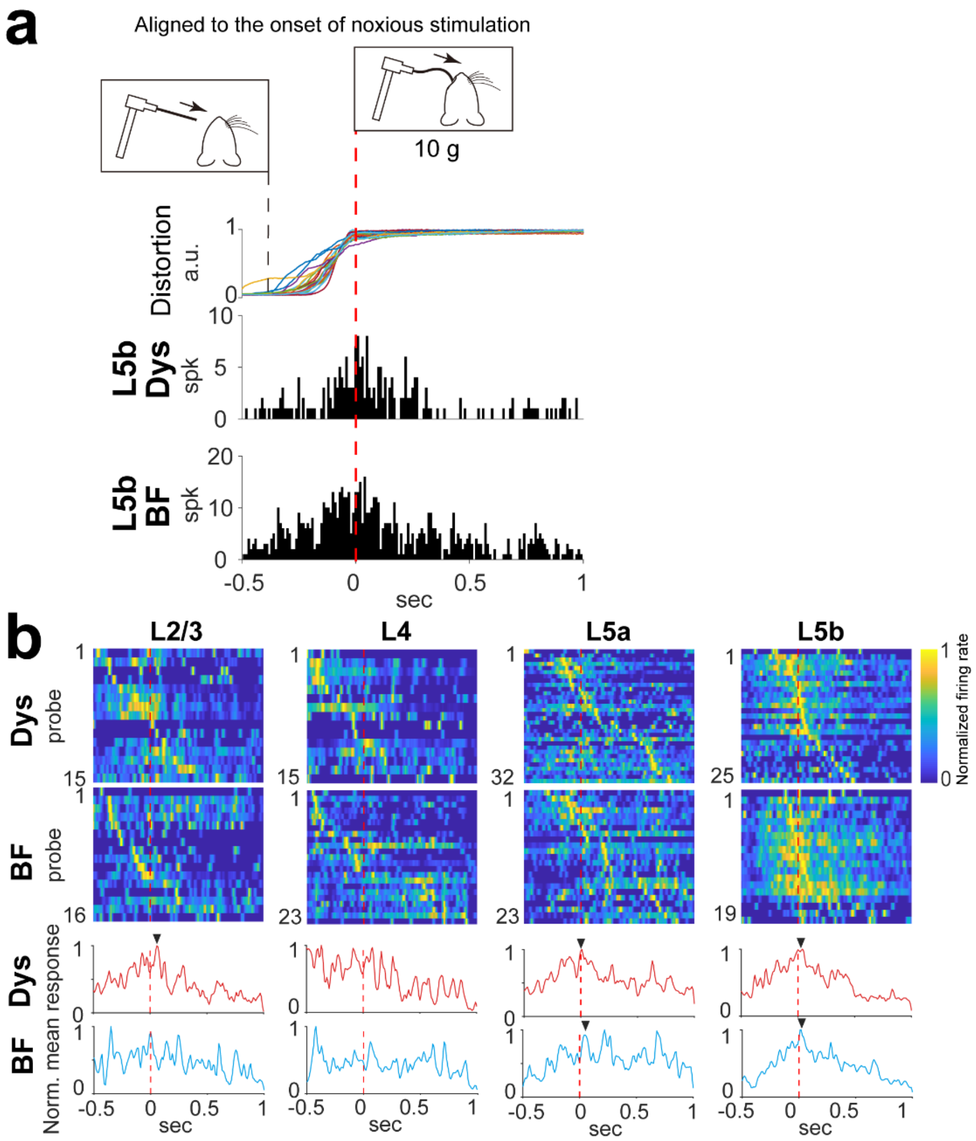


**Supplementary Fig. 5**

**Neurons in deeper layers respond to noxious mechanical stimuli on the whisker pad**

**a**, Example MUA response to a noxious mechanical stimulus. *Upper*, The onset of the response to a 10-g von Frey filament (red dotted line) was detected by a high-speed camera monitoring distortion of the left whisker pad (colored lines, normalized mean response, see Methods). *Lower*, PSTHs of MUA in Dys and BF L5b aligned to the onset of the von Frey filament. **b**, *Upper*, Normalized MUA recorded from each probe in each layer (n = 5 animals). *Lower*, Normalized mean responses of Dys (red) and BF (blue).

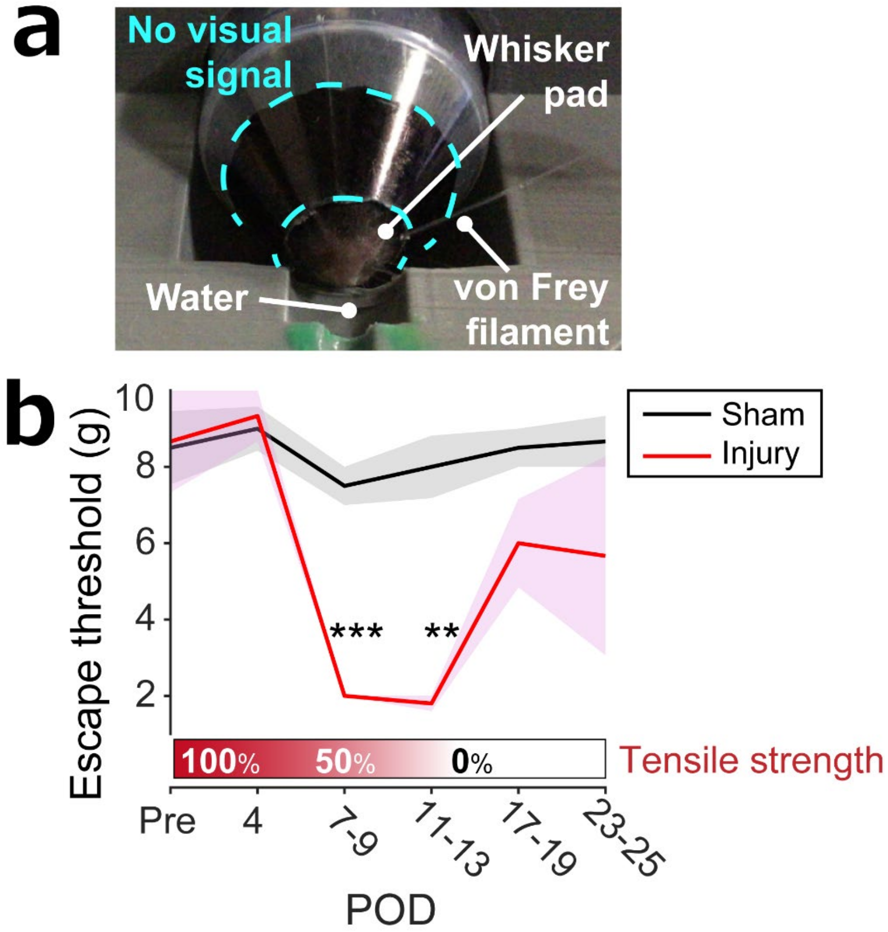


**Supplementary Fig. 6**

**Infraorbital nerve ligation by absorbable surgical thread-induced allodynia**

**a**, Setup for the von Frey test. **b**, Mechanical allodynia was observed at 7–9 (***$P$ = 0.0002) and 11–13 (**$P$ = 0.0015) days after the nerve ligation (one-way ANOVAs followed by Dunnett's test). Error bars and shading indicate SEMs. Sham, $n$ = 3; injury, $n$ = 3.

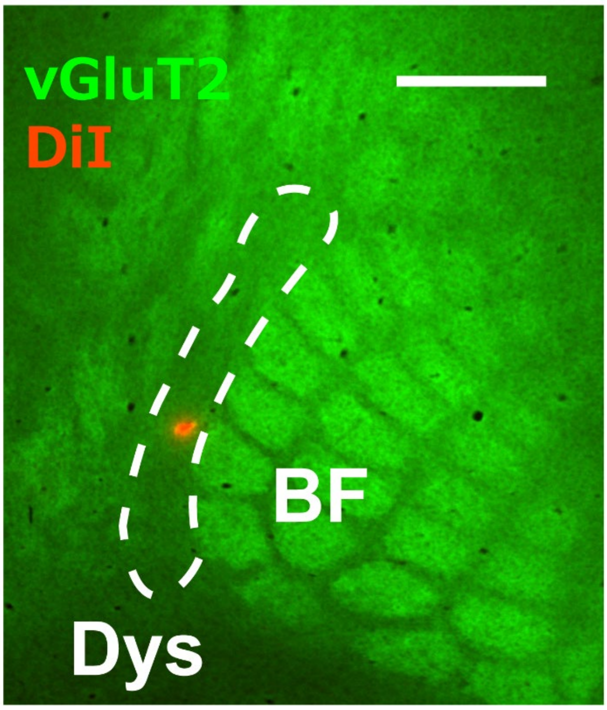


**Supplementary Fig. 7**

**Region adjacent to BF was identified as Dys**

An electrode stained with DiI (red) was inserted into the region adjacent to BF after intrinsic signal imaging was performed. Immunohistochemistry was performed on a tangential section of L4 to identify BF via VGluT2 (green). Scale bar, 500 µm.

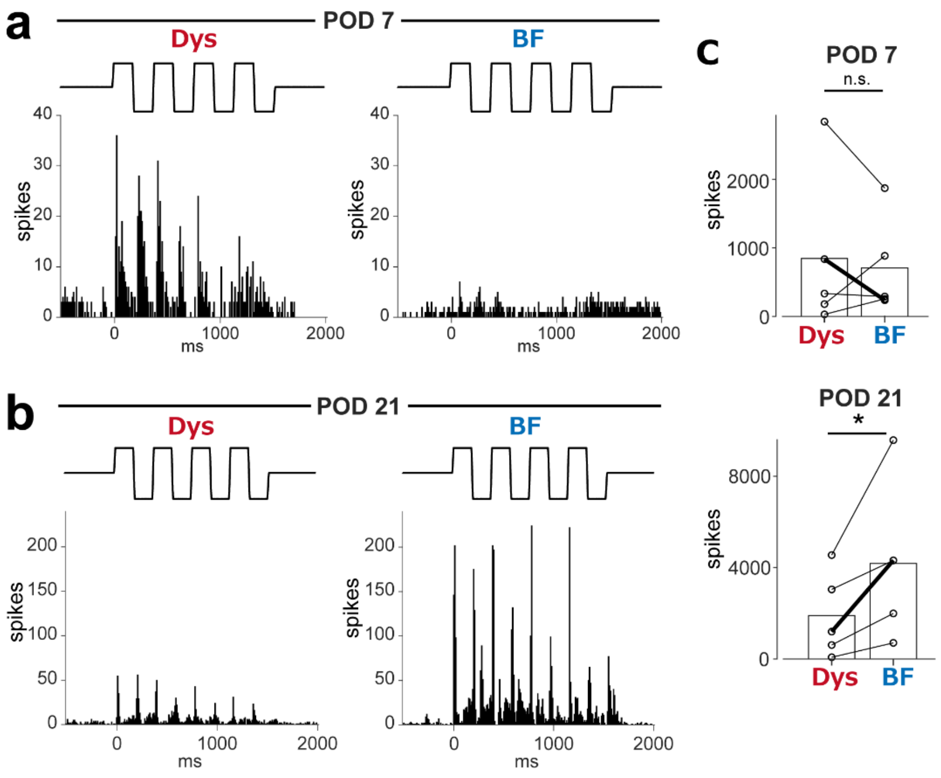


**Supplementary Fig. 8**

**Infraorbital nerve ligation by absorbable surgical thread changed the balance of activity between Dys and BF**

**a**, At POD7, Dys L4 neurons were more activated by whisker deflection than were BF L4 neurons. **b**, At POD21, neuronal activity in BF L4 was recovered and higher than in Dys L4. **c**, Total spike numbers of the MUA of L4 neurons in Dys and BF at POD7 and POD21 during tactile stimulation (from the onset of the stimulus to 1500 msec) (n = 5 animals for each group; **P* = 0.046, two-sided paired-sample *t*-test). Each bar indicates the mean of each group. Each connected point indicates pairs recorded from the same animal. Thick lines indicate the example pair shown in a and b.

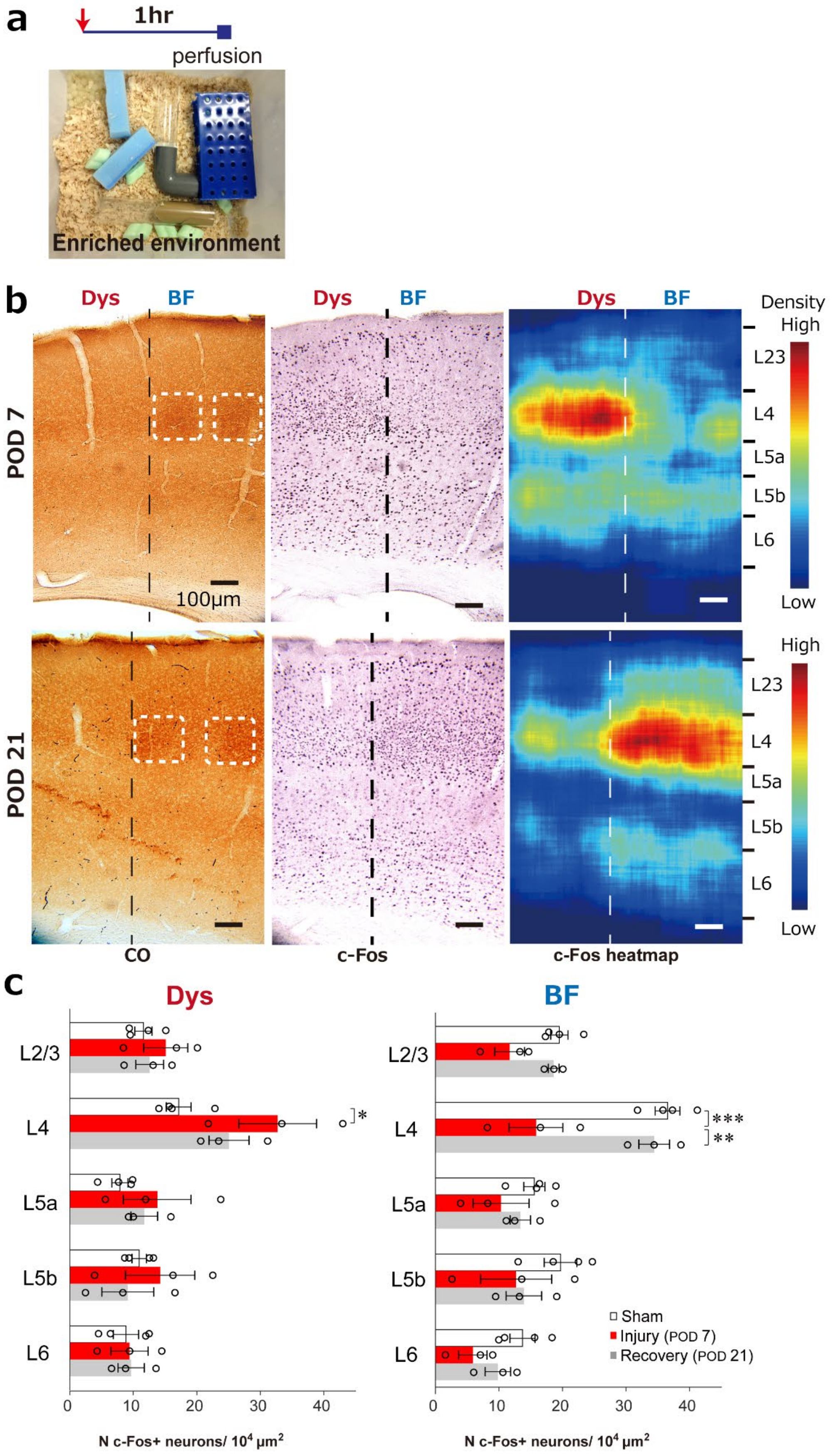
a
1hr
perfusion
Enriched environment
b
Dys
BF
Density
High
Low
L23
L4
L5a
L5b
L6
POD 7
POD 21
100μm
CO
c-Fos
c-Fos heatmap
c
Dys
BF
L2/3
L4
L5a
L5b
L6
*
***
**
Sham
Injury (POD 7)
Recovery (POD 21)
0
10
20
30
40
N c-Fos+ neurons/ 10⁴ μm²

**Supplementary Fig. 9**

**Infraorbital nerve ligation by absorbable surgical thread-activated Dys under an enriched environment and induced allodynia**

**a-c**, Dys was activated under enriched environmental conditions during infraorbital nerve ligation. **a**, Animals were placed in a novel and enriched environment for 1 h before perfusion. **b**, Examples of cytochrome oxidase (CO) staining, c-Fos expression, and a c-Fos density heat map at POD7 and POD21. **c**, L4 of Dys was activated at POD7. c-Fos expression in BF L4 decreased at POD7 and recovered to sham level at POD21. Data are presented as mean values ± SEM. *$P$ = 0.0485, **$P$ = 0.00318, ***$P$ = 0.000242 by one-way ANOVA followed by the Tukey–Kramer test; sham, n = 4; POD7, n = 3; POD21, n = 3.

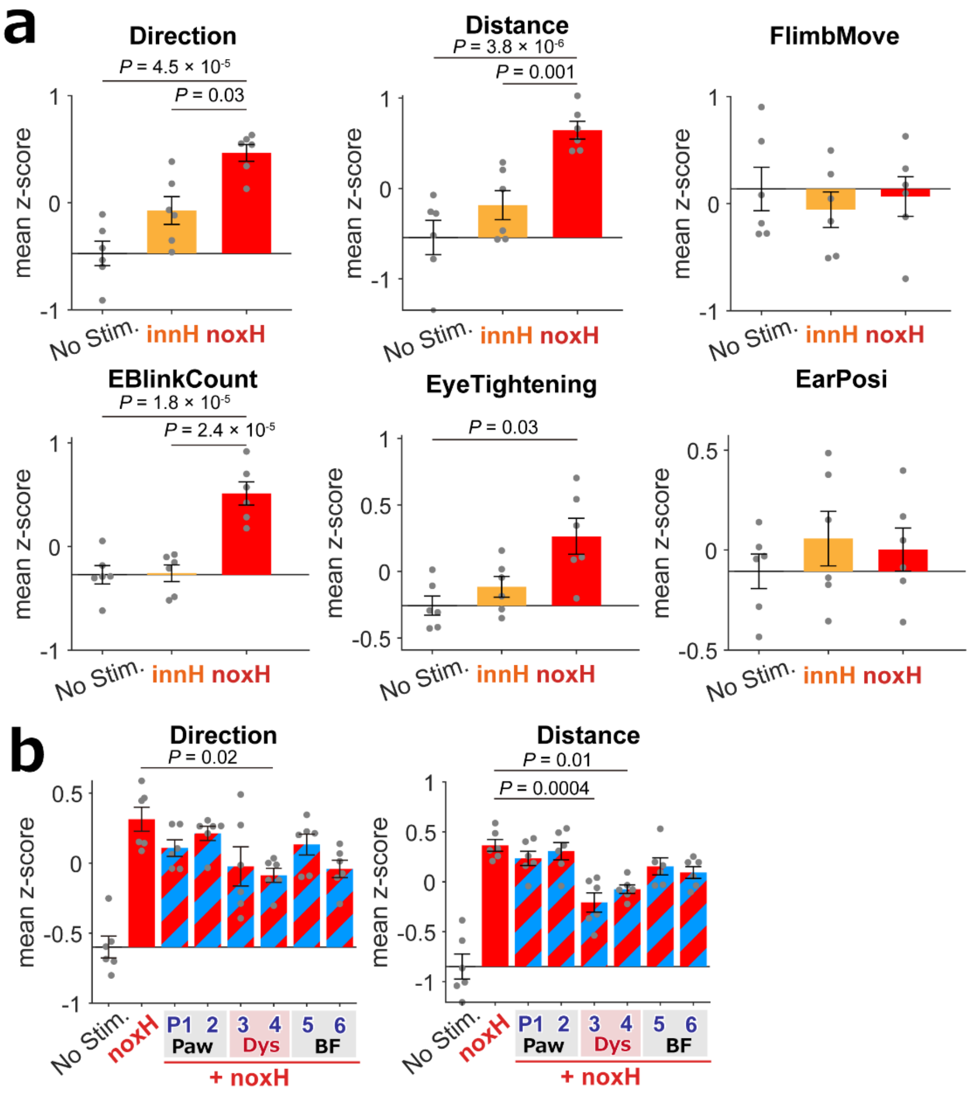


**Supplementary Fig. 10**

**Nocifensive responses taken from behavioural experiments using a spherical treadmill**

**a**, Indices to measure nocifensive responses to IR laser exposure for 0 (no stim), 500 (innH), and 1,500 ms (noxH). Direction, the moving direction; a high z-score indicates the animal moved contralateral to the direction of the IR laser. Distance, the total distance traveled 4 s after the onset of IR laser. FlimbMove, the number of touches or covering of the left whisker pad by the left forelimb. EyeBlink, the number of eye blinks. Eye tightening, sustained closure of the left eyelid. EarPosi, the position of the lower edge of the ear lobe. Error bars indicate SEMs (*n* = 6 animals). P-values were calculated by one-way ANOVA followed by the Tukey–Kramer test. Error bars indicate SEMs.**b**, Study of the activation of PV interneurons in different anatomical positions (*n* = 6 animals). P-values were calculated by one-way ANOVA followed by the Tukey–Kramer test. Error bars indicate SEMs.

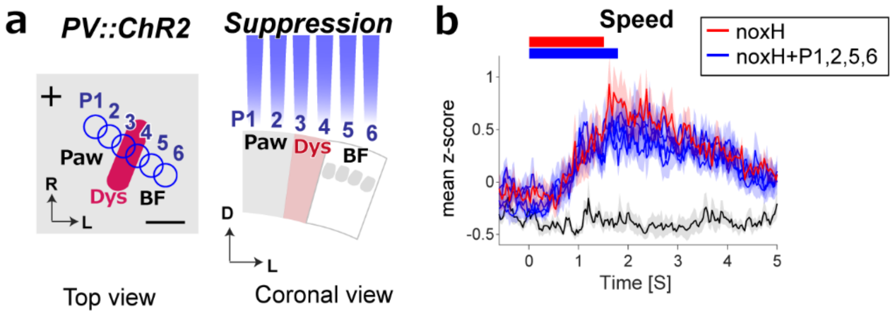


**Supplementary Fig. 11**

**Blue light stimulation of BF did not reduce escape speed**

**a**, Same setup as shown in Fig. 4e. **b**, Blue light stimulation of P1, 2, 5, and 6 did not reduce the escape speed to noxH. The difference between the IR-only condition and P1, 2, 5, 6 activation combined with IR stimulation was statistically insignificant at all time points (one-way ANOVA followed by Tukey–Kramer test, n = 6 animals). Line and shading indicate mean ± SEM.

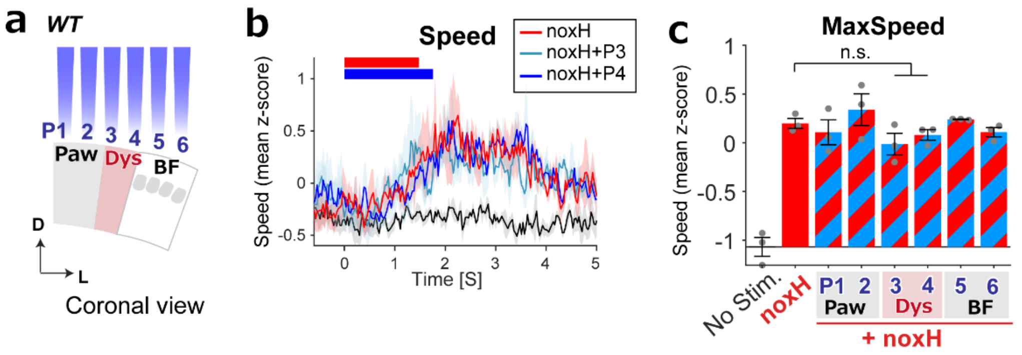


**Supplementary Fig. 12**

**Blue light stimulation of Dys in control animals did not reduce escape speed**

**a**, Same setup as shown in Fig. 4e but ChR2-negative wild-type (WT) animals. **b**, Blue light stimulations of P3 and P4 did not reduce the escape speed in WT mice ($n$ = 3 animals). Line and shading indicate mean ± SEM. **c**, Blue light stimulation at any position did not affect MaxSpeed (one-way ANOVA followed by Tukey–Kramer test, n = 3 animals). Error bars indicate SEMs.

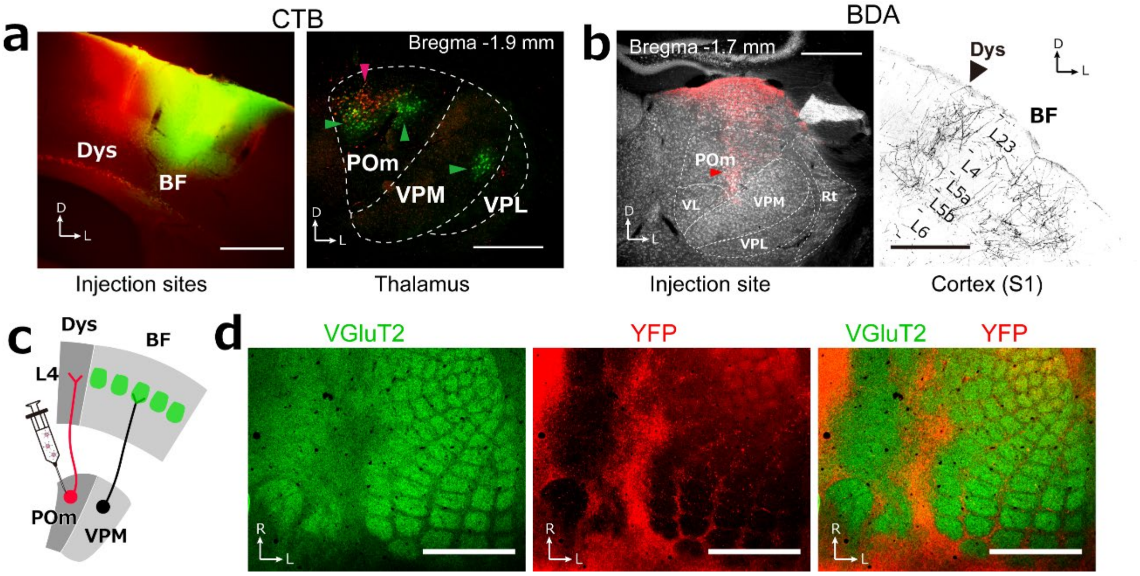


**Supplementary Fig. 13**

**Dys receives input from the posterior medial thalamic nucleus**

**a**, Retrograde labeling of posterior medial thalamic nucleus (POm) neurons after the injection of Alexa Fluor 555- and 488-labeled cholera toxin subunit B into Dys (red) and BF (green), respectively. *Left*, Injection sites. *Right*, Somas of neurons projecting to Dys were observed in POm. The somas of neurons projecting to BF were observed in the ventral posterior medial nucleus (VPM) and POm. VPL, ventral posterior lateral nucleus. Scale bars, 500 µm. **b**, *Left*, Injection site of biotinylated dextran amine (BDA), an anterograde tracer. *Right*, Axon terminals from POm neurons. VL, ventral lateral nucleus; Rt, reticular nucleus. Scale bars, 500 µm. **c**, Schema of the AAV-DJ-YFP injection. **d**, Tangential section of S1 L4 co-labelled with anti-VGluT2 antibody (green) and axon terminals from POm (red). Scale bars, 1 mm. See also other tracer studies[2,3].

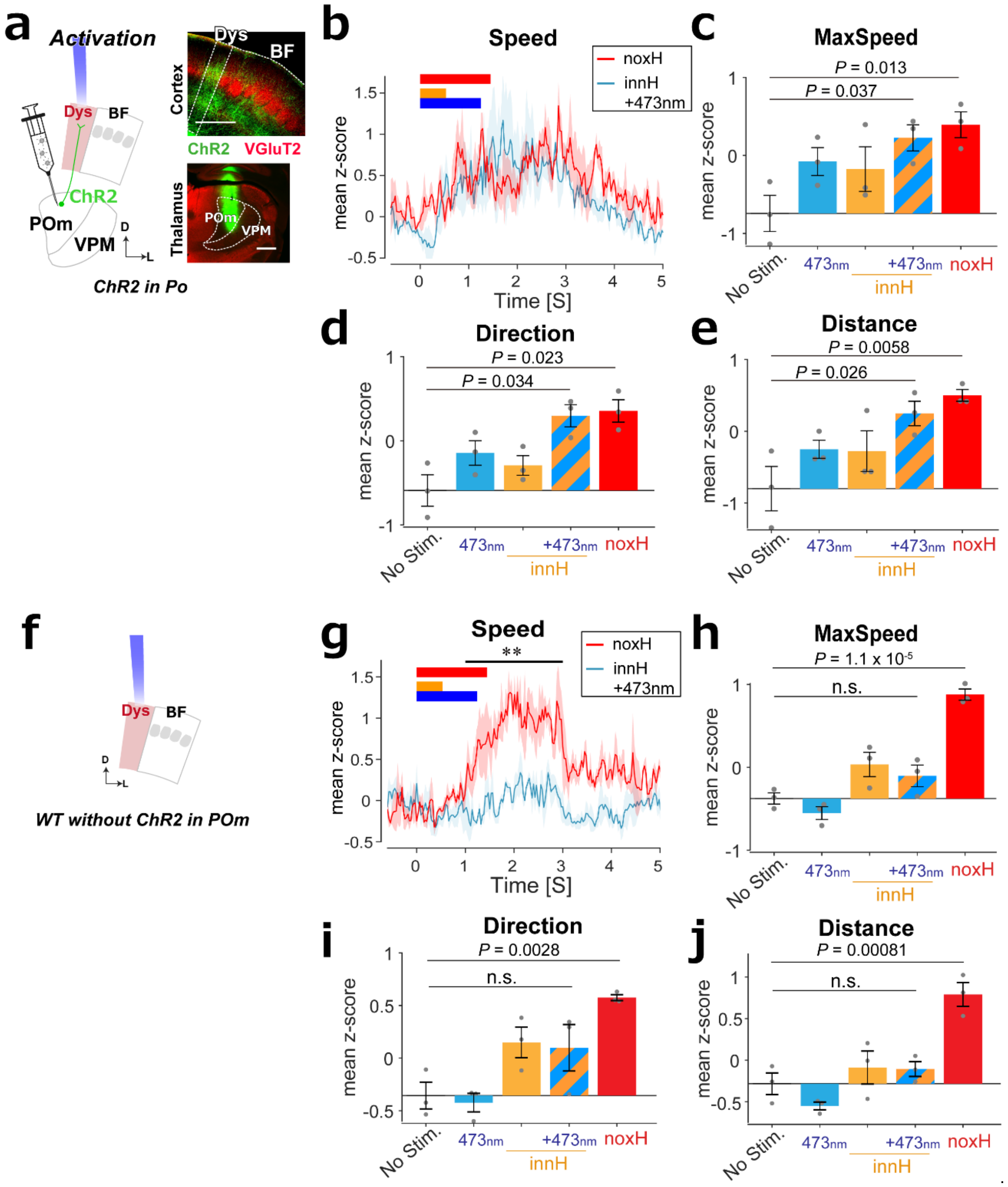


**Supplementary Fig. 14**

**Optogenetical activation of thalamocortical fibers projecting to Dys induced escape responses**

**a**, Scheme for activating thalamocortical fibers from the posterior medial thalamic nucleus (POm) and an example of ChR2 expression in POm and in thalamocortical fibres in Dys. VPM, ventral posterior medial nucleus. Scale bars, 500 µm. **b**, The average speed profile for optogenetic activation with innH matched that for noxH ($n$ = 3 animals). Shading indicates SEMs for mice.

Red bar, the time of IR laser irradiation for noxH. Orange bar, the time of IR laser irradiation for innH. Blue bar, the time of the 473-nm laser irradiation. **c**, Mean z-scores of the maximum speed (n = 3 animals). **d**, Mean z-scores of the running direction (n = 3 animals). **e**, Mean z-scores of the distance (n = 3 animals). **f-k**, Same setup as a in WT mice without AAV ChR2 injection into POm. **g**, Mean z-scores of the speed profiles under the noxH condition and innH + 473 nm stimulation. Shading indicates SEM over three mice. Color bars are the same as in b. **h**, Mean z-scores for maximum speed (n = 3 animals). **i**, Mean z-scores of the running direction (n = 3 animals). **j**, Mean z-scores of the distance (n = 3 animals). The data of No Stim., innH, and noxH without 473 nm sessions are the same as shown in Fig. 4d and Supplementary Fig. 7a. The blue light with/without IR stimulation did not increase MaxSpeed. n.s., not significant; $^{**}P < 0.01$; one-way ANOVA followed by the Tukey–Kramer test. Error bars indicate SEMs.

## Supplementary References

1 Benedetti, B. L., Glazewski, S. & Barth, A. L. Reliable and Precise Neuronal Firing during Sensory Plasticity in Superficial Layers of Primary Somatosensory Cortex. *Journal of Neuroscience* **29**, 11817-11827, doi:10.1523/jneurosci.3431-09.2009 (2009).

2 Wimmer, V. C., Bruno, R. M., de Kock, C. P. J., Kuner, T. & Sakmann, B. Dimensions of a projection column and architecture of VPM and POm axons in rat vibrissal cortex. *Cerebral cortex (New York, N.Y. : 1991)* **20**, 2265-2276, doi:10.1093/cercor/bhq068 (2010).

3 Koralek, K.-A., Jensen, K. F. & Killackey, H. P. Evidence for two complementary patterns of thalamic input to the rat somatosensory cortex. *Brain Research* **463**, 346-351, doi:10.1016/0006-8993(88)90408-8 (1988).
