# Supplementary material for: Distinct nociception processing in the dysgranular and barrel regions of the mouse somatosensory cortex
Source: Nat Commun. 2022 Jun 29;13:3622. doi: 10.1038/s41467-022-31272-w (PMC9243138; doi:10.1038/s41467-022-31272-w)
Supplement: Supplementary file 3 — Description of Additional Supplementary Files [file 41467_2022_31272_MOESM3_ESM.pdf]

Description of Additional Supplementary Files

Supplementary Software 1

The software 'MouseDetectionTrackBall_VariableIRlength.m' was used to monitor the animal behavior in response to infrared laser to the left whisker pad.

Supplementary Software 2

The softwares 'CfosCounter.m' and 'CfosCounter.fig' were used to count the expression of c-Fos for bright field observations (Supplementary Fig. 9).
