# Supplementary figures and images for: Distinct nociception processing in the dysgranular and barrel regions of the mouse somatosensory cortex
Source: Nat Commun. 2022 Jun 29;13:3622. doi: 10.1038/s41467-022-31272-w (PMC9243138; doi:10.1038/s41467-022-31272-w)

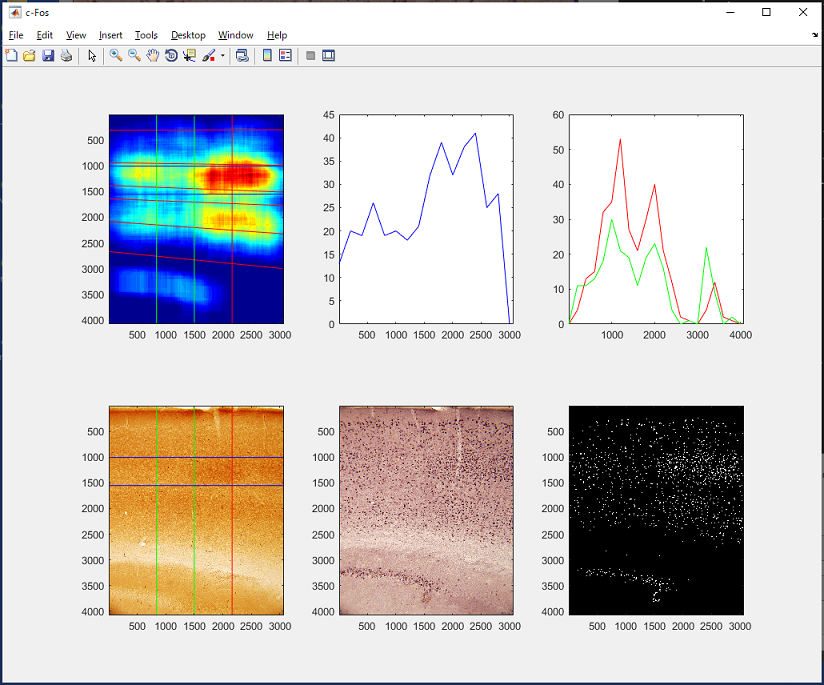

Supplement: Supplementary file 4 — Supplementary Software 1 [file 41467_2022_31272_MOESM4_ESM.zip › Supplementary Software 1/ExportedFig.png]

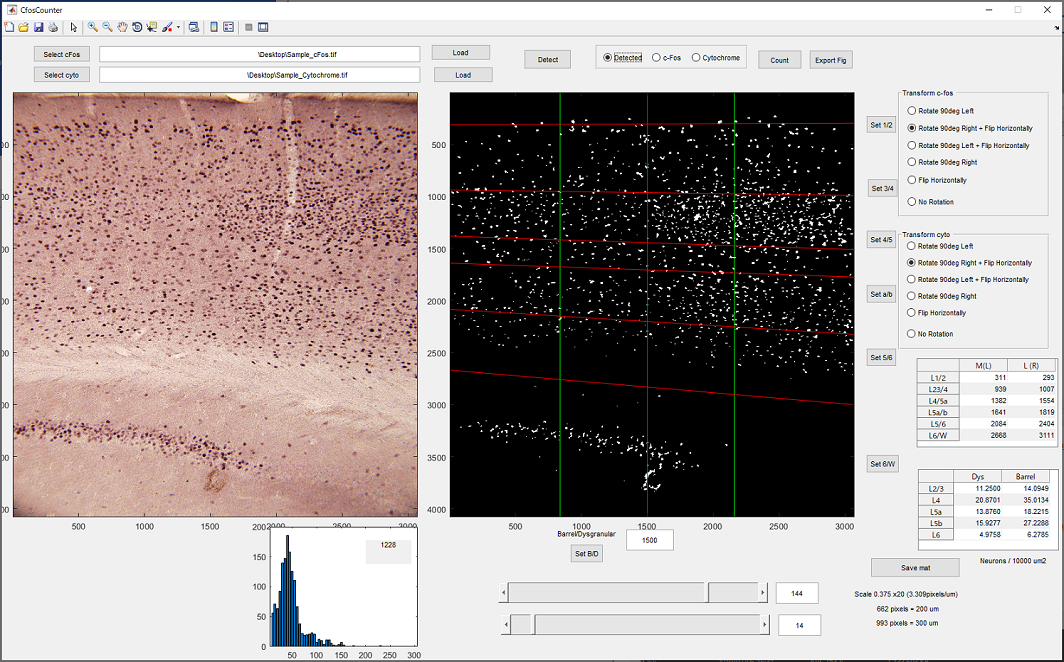

Supplement: Supplementary file 4 — Supplementary Software 1 [file 41467_2022_31272_MOESM4_ESM.zip › Supplementary Software 1/Sample.png]

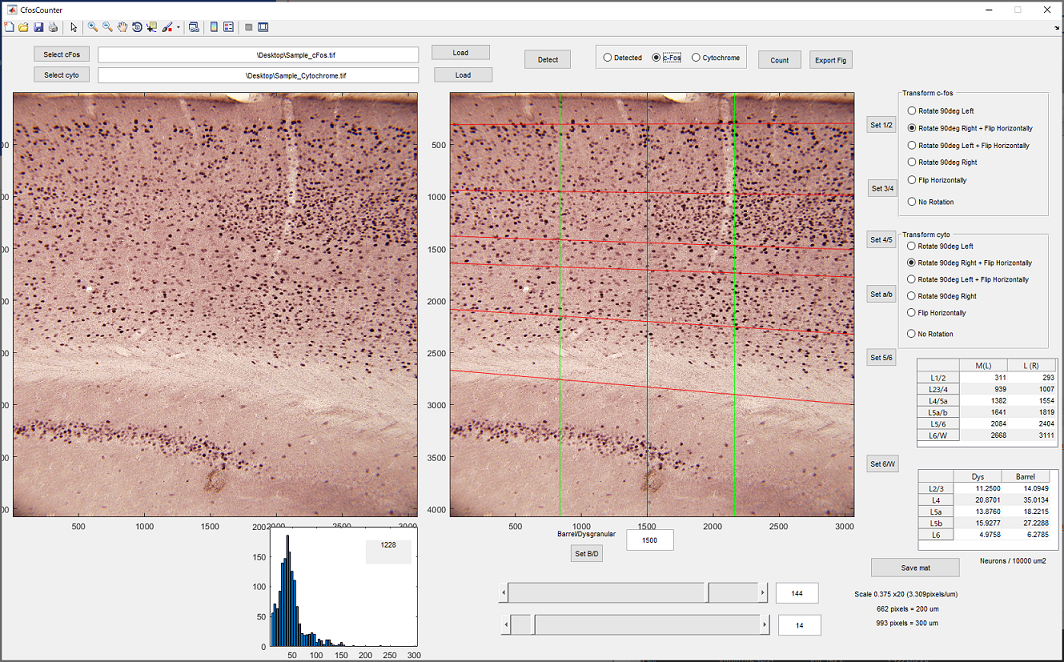

Supplement: Supplementary file 4 — Supplementary Software 1 [file 41467_2022_31272_MOESM4_ESM.zip › Supplementary Software 1/Sample2.png]

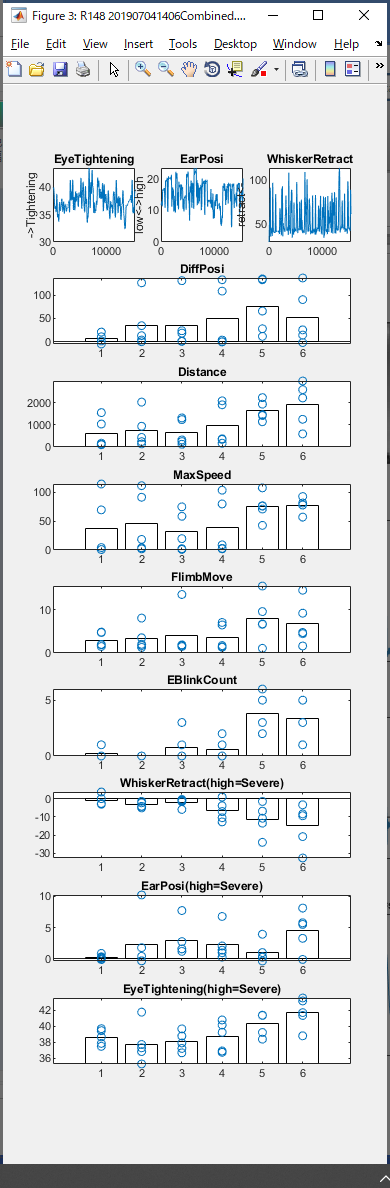

Supplement: Supplementary file 5 — Supplementary Software 2 [file 41467_2022_31272_MOESM5_ESM.zip › Supplementary Software 2/BarGraph.png]

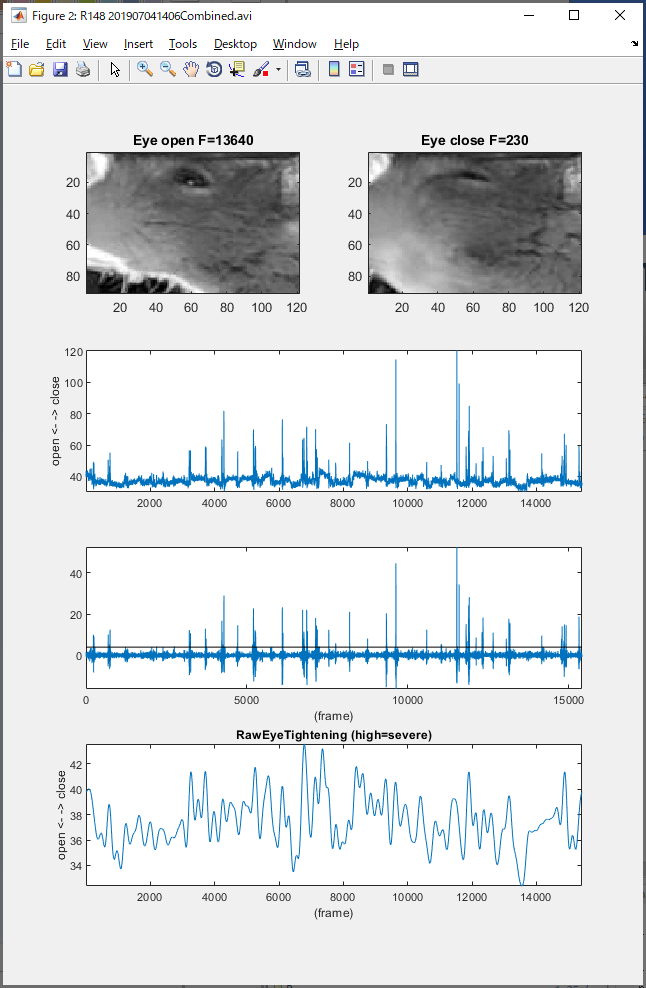

Supplement: Supplementary file 5 — Supplementary Software 2 [file 41467_2022_31272_MOESM5_ESM.zip › Supplementary Software 2/Eye.png]

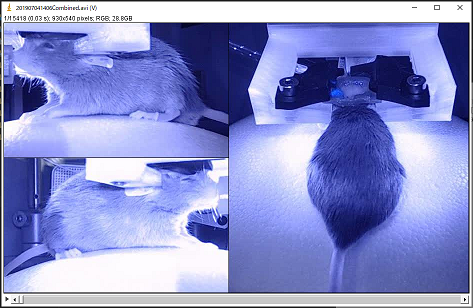

Supplement: Supplementary file 5 — Supplementary Software 2 [file 41467_2022_31272_MOESM5_ESM.zip › Supplementary Software 2/SampleFrame.png]

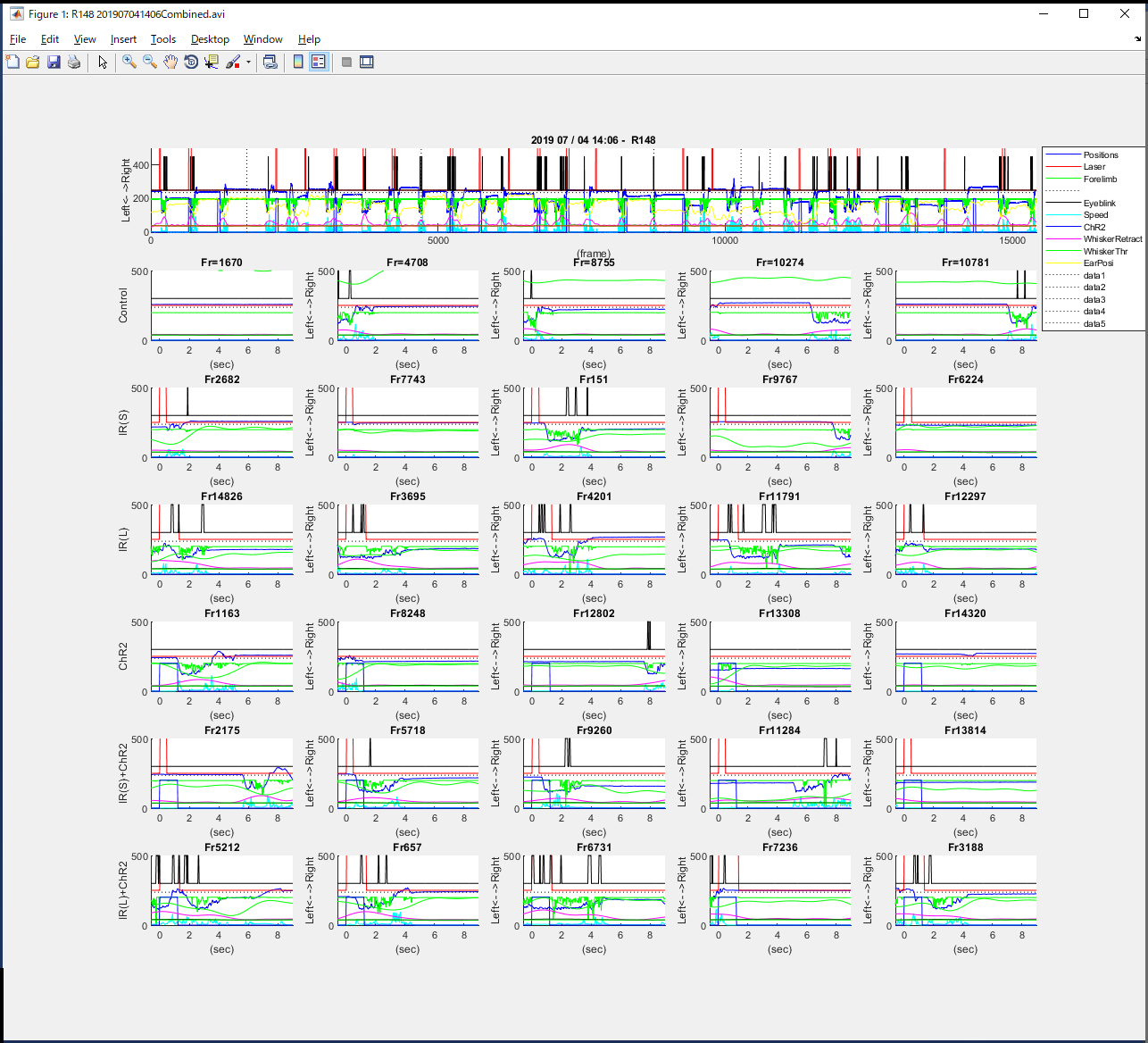

Supplement: Supplementary file 5 — Supplementary Software 2 [file 41467_2022_31272_MOESM5_ESM.zip › Supplementary Software 2/Samples_Alltrialdata.png]
