# Supplementary material for: Distinct nociception processing in the dysgranular and barrel regions of the mouse somatosensory cortex
Source: Nat Commun. 2022 Jun 29;13:3622. doi: 10.1038/s41467-022-31272-w (PMC9243138; doi:10.1038/s41467-022-31272-w)
Supplement: Supplementary file 6 — Reporting Summary [file 41467_2022_31272_MOESM6_ESM.pdf]

nature portfolio

Corresponding author(s): Hironobu Osaki and Mariko Miyata

Last updated by author(s): May 20, 2022

Reporting Summary

Nature Portfolio wishes to improve the reproducibility of the work that we publish. This form provides structure for consistency and transparency in reporting. For further information on Nature Portfolio policies, see our Editorial Policies and the Editorial Policy Checklist.

Statistics

For all statistical analyses, confirm that the following items are present in the figure legend, table legend, main text, or Methods section.

n/a	Confirmed	
☐	☒	The exact sample size (n) for each experimental group/condition, given as a discrete number and unit of measurement
☐	☒	A statement on whether measurements were taken from distinct samples or whether the same sample was measured repeatedly
☐	☒	The statistical test(s) used AND whether they are one- or two-sided<br>*Only common tests should be described solely by name; describe more complex techniques in the Methods section.*
☒	☐	A description of all covariates tested
☐	☒	A description of any assumptions or corrections, such as tests of normality and adjustment for multiple comparisons
☐	☒	A full description of the statistical parameters including central tendency (e.g. means) or other basic estimates (e.g. regression coefficient) AND variation (e.g. standard deviation) or associated estimates of uncertainty (e.g. confidence intervals)
☐	☒	For null hypothesis testing, the test statistic (e.g. F, t, r) with confidence intervals, effect sizes, degrees of freedom and P value noted<br>*Give P values as exact values whenever suitable.*
☒	☐	For Bayesian analysis, information on the choice of priors and Markov chain Monte Carlo settings
☒	☐	For hierarchical and complex designs, identification of the appropriate level for tests and full reporting of outcomes
☒	☐	Estimates of effect sizes (e.g. Cohen's d, Pearson's r), indicating how they were calculated

Our web collection on statistics for biologists contains articles on many of the points above.

Software and code

Policy information about availability of computer code

Data collection	Images were captured with upright microscopes with a cooled-CCD camera with µManager (ver. 1.4, https://micro-manager.org/) or with a CCD camera using Olympus DP Manager (ver. 3.1.1.208, Olympus).<br>The spike sorting comprised automated spike detection and clustering using Klusta (ver. 3.0.16) followed by manual sorting using Kwik GUI (v1.0.9), or using Kilosort (v2.0, https://github.com/cortex-lab/KiloSort) followed by manual sorting using Phy (v2.0b1, https://phy.readthedocs.io/en/latest/).<br>For intrinsic signal optical imaging, the images from a CMOS camera were acquired by microDisplay software (ver. 5.2.3.1, Mannheim, Germany).<br>Tactile and heat stimuli for electrophysiological recording or intrinsic signal optical imaging were generated by custom-written Matlab code with data acquisition toolbox (Mathworks, version: 2015a) .<br>All stimulation trials by a von Frey filament during electrophysiological recordings were monitored by a high-speed camera recording at 200 Hz (XiQ, Ximea GmbH, Münster, Germany).<br>For controlling stimuli during animal behavioural study on spherical treadmill, custom-written Matlab code with data acquisition toolbox (2018a) was used.<br>Animal behaviour was recorded using three CMOS cameras controlled by MATLAB (2018a) with image acquisition toolbox.<br>The versions of Matlab depend on the computers and connected hardwares.
Data analysis	Counting c-Fos positive neurons was performed using custom-written MATLAB code with image processing toolbox.<br>For electrophysiological recording data, Klusta and Kwik-gui (Rossant, C. et al., 2016) were used for automated spike sorting followed by manual sorting.<br>After manual sorting, custom-written Matlab code with signal processing toolbox was used for analysis spiking neural activity after manual sorted neurons, such as calculating peristimulus time histogram and signal to noise ratio.<br>For intrinsic signal optical imaging, custom written Matlab code with image processing toolbox (2016b) was used to determine the signal areas by edge detection methods.<br>For analysis of animal behaviour on spherical treadmill, custom written Matlab code with image processing toolbox (2018a) was used.

Statistical tests were performed in Matlab with statistics and machine learning toolbox.
All custom-written code is available from the corresponding authors upon reasonable request.

For manuscripts utilizing custom algorithms or software that are central to the research but not yet described in published literature, software must be made available to editors and reviewers. We strongly encourage code deposition in a community repository (e.g. GitHub). See the Nature Portfolio guidelines for submitting code & software for further information.

Data

Policy information about availability of data

All manuscripts must include a data availability statement. This statement should provide the following information, where applicable:
- Accession codes, unique identifiers, or web links for publicly available datasets
- A description of any restrictions on data availability
- For clinical datasets or third party data, please ensure that the statement adheres to our policy

All data supporting the findings of this study are provided within the paper and its Supplementary information. A Source data file is provided with this paper. Any data are available from the authors upon request.

Field-specific reporting

Please select the one below that is the best fit for your research. If you are not sure, read the appropriate sections before making your selection.

☒ Life sciences ☐ Behavioural & social sciences ☐ Ecological, evolutionary & environmental sciences

For a reference copy of the document with all sections, see nature.com/documents/nr-reporting-summary-flat.pdf

Life sciences study design

All studies must disclose on these points even when the disclosure is negative.

Sample size	Sample sizes (number of neurons, sections, animals, and sessions for stimulation during recording) were approximated based on previous studies (Fukui et al., 2020, Sato et al., 2019).
Data exclusions	For animal behavioral study on a spherical treadmill, the session was excluded from the analysis when the animal ran continuously across sessions on the treadmill: If the animal ran continuously on the treadmill prior to the onset of the stimulus, the effect of the stimulus was masked. These animals tended to run continuously regardless of the stimuli. Therefore, no difference in the maximum speed between trials could be observed. The criteria was established before the recording sessions, in which optogenetics was used to modulate animal behavior.
Replication	All experiments were replicated successfully between animals independently at least three times or with the same number of animals shown in each experiment. The number of animals used in the experiments is stated in the paper.
Randomization	The stimulus types (i.e. length of infrared laser and position of 473nm optogenetics stimulation) used in behavioural studies were randomly chosen in each session.<br>Animals were assigned to either ligation of infraorbital nerve or sham operation group randomly.
Blinding	The cFos manual cell counting in Supplementary Fig. 1 was performed by a person who was not involved in the injection of capsaicin and vehicle. For the cFos cell counting in Supplementary Fig. 8, the number of cFos positive cells was counted automatically by custom-written Matlab code with image processing toolbox.<br>For intrinsic signal optical imaging during ligation, the experimenter was not blind during imaging since the signal strength and the signal area were clearly different after ligation and sham operation.<br>For von Frey test, the experimenter was blind to the ligation or the sham group during the data acquisition. During data analysis for von Frey test, blinding was not relevant to data analysis because comparing values between the groups was not affected by blinding.

Reporting for specific materials, systems and methods

We require information from authors about some types of materials, experimental systems and methods used in many studies. Here, indicate whether each material, system or method listed is relevant to your study. If you are not sure if a list item applies to your research, read the appropriate section before selecting a response.

Materials & experimental systems

n/a	Involved in the study
☐	☒ Antibodies
☒	☐ Eukaryotic cell lines
☒	☐ Palaeontology and archaeology
☐	☒ Animals and other organisms
☒	☐ Human research participants
☒	☐ Clinical data
☒	☐ Dual use research of concern

Methods

n/a	Involved in the study
☒	☐ ChIP-seq
☒	☐ Flow cytometry
☒	☐ MRI-based neuroimaging

Antibodies

Antibodies used

All of the antibodies listed here described in the Methods.
The primary antibodies:
anti-c-Fos antibody (rabbit; 1:10000, SAB5700610-100UL, Merck KGaA, Darmstadt, Germany)
anti-c-Fos antibody (rabbit; 1:2000, 226 003, Synaptic Systems GmbH, Göttingen, Germany)
anti VGluT2 antibody (guinea pig; 1:500, MSFR106290, Nittobo Medical Co., Ltd., Tokyo, Japan)
a goat polyclonal antibody against calbindin D-28K (goat; 1:500, MSFR100410, Nittobo Medical Co., Ltd.)
a mouse monoclonal antibody against NeuN (mouse; 1:500, MAB377, Merck, Darmstadt, Germany)

The secondary antibodies:
Alexa Fluor 647 conjugated-anti-guinea pig antibody (donkey; 1:500, 706-605-148, Jackson ImmunoResearch, West Grove, PA, USA)
biotinylated goat anti-rabbit IgG antibody (goat; 1:200, BA-1000-1.5, Vector Laboratories, Burlingame, CA, USA)
Alexa Fluor Plus 405-conjugated Donkey anti-Mouse IgG antibody (Donkey; 1:500, A48257, Thermo Fisher Scientific)
Alexa Fluor 488-conjugated Donkey anti-Goat IgG antibody (Donkey; 1:500, A11055, Thermo Fisher Scientific)
Alexa Fluor Plus 555-conjugated Donkey anti-Rabbit IgG antibody (Donkey; 1:500, A32794, Thermo Fisher Scientific, Waltham, MA, USA)
Alexa Fluor 594-conjugated anti-guinea pig antibody (Donkey; 1:500, 706-585-148, Jackson ImmunoResearch)

Validation

All of the antibodies used in our study are commercially available.

For rabbit c-Fos antibody, Merck KGaA provides validation information as follows:
"Evaluated by Western Blotting in PMA(TPA) treated HeLa cell lysate.
Western Blotting Analysis: 0.5 µg/mL of this antibody detected c-Fos in 10 µg of PMA(TPA) treated HeLa cell lysate."

For rabbit c-Fos antibody, Synaptic Systems GmbH provides validation information as follows: ""Immunogen: Synthetic peptide corresponding to AA 2 to 17 from rat c-Fos (UniProt Id: P12841). Reacts with: human (P01100), rat (P12841), mouse (P01101), monkey, ape, cow,dog, pig.Other species not tested yet."

For vGluT2 antibody, Frontier Institute (Manufacture) provides validation information as follows:
"mouse (others not tested). Immunoblot detects a single protein band at 60 kDa. This selectively stains distinct populations of glutamatergic neurons, particularly their terminals."

For calbindin antibody, Frontier Institute (Manufacture) provides validation information as follows: "mouse (others not tested). Immunoblot detects a single protein band at 28 kDa."

For antibody against NeuN, Merk provides validation information as follows: "Vertebrate neuron-specific nuclear protein called NeuN (Neuronal Nuclei). Only one NeuN clone exists (A60) and reacts with an uncharacterized nuclear protein. MAB377 reacts with most neuronal cell types throughout the nervous system of mice including cerebellum, cerebral cortex, hippocampus, thalamus, spinal cord and neurons in the peripheral nervous system including dorsal root ganglia, sympathetic chain ganglia and enteric ganglia. The immunohistochemical staining is primarily in the nucleus of the neurons with lighter staining in the cytoplasm. The few cell types not reactive with MAB377 include Purkinje, mitral and photoreceptor cells. Developmentally, immunoreactivity is first observed shortly after neurons have become postmitotic, no staining has been observed in proliferative zones. The antibody is an excellent marker for neurons in primary cultures and in retinoic acid-stimulated P19 cells. It is also useful for identifying neurons in transplants."

Animals and other organisms

Policy information about studies involving animals; ARRIVE guidelines recommended for reporting animal research

Laboratory animals

The following mouse lines were used in this study: C57BL/6N (Sankyo Lab. Service Corp., Tokyo, Japan), PV-cre(B6;129P2-Pvalbtm1(cre)Arbr/J ,JAX stock #008069), Ai32 (Rosa-CAG-LSL-ChR2[H134R]-EYFP-WPRE; JAX stock #012569). Male mice of 8 weeks or older in age were used.

Wild animals

No wild animals were used in this study.

Field-collected samples

No field-collected samples were in this study.

Ethics oversight	All protocols and procedures followed the guidelines of the Animal Care and Use Committee of Tokyo Women's Medical University.

Note that full information on the approval of the study protocol must also be provided in the manuscript.
